# “A secret club”: focus groups about women’s toileting behaviors

**DOI:** 10.1186/s12905-019-0740-3

**Published:** 2019-03-07

**Authors:** Mary H. Palmer, Jennifer M. Wu, Celine S. Marquez, Betty Rupp, Mitchell M. Conover, Diane K. Newman

**Affiliations:** 1 Helen W. & Thomas L. Umphlet Distinguished Professor in Aging, School of Nursing, University of North Carolina at Chapel Hill, CB 7460, Chapel Hill, NC 27599-7460 USA; 2 Department of Obstetrics and Gynecology, Division of Urogynecology and Reconstructive Pelvic Surgery, School of Medicine, University of North Carolina at Chapel Hill, 3032 Old Clinic Building, CB#7570, Chapel Hill, NC 27599-7570 USA; 3 School of Nursing, University of North Carolina at Chapel Hill, CB#7460, Chapel Hill, NC 27599-7460 USA; 4 UNC Dept. of Biostatistics, Collaborative Studies Coordinating Center, 123 W. Franklin St., Ste. 450, CB #8030, Chapel Hill, NC 27514 USA; 5 Department of Epidemiology, School of Global Public Health, University of North Carolina at Chapel Hill, Gillings, Chapel Hill, NC 27599-7435 USA; 6 Perelman School of Medicine, University of Pennsylvania, 3rd floor, West Pavilion, Perelman Center 34th & Civic Center Blvd, Philadelphia, PA 19104 USA

**Keywords:** Female, Urination, Urinary incontinence, Cues, Awareness

## Abstract

**Background:**

Understanding reasons for and impact of women’s toileting behaviors on bladder health is important to prevent and manage urinary incontinence (UI) and overactive bladder (OAB).

**Methods:**

Women, regardless of urinary incontinence (UI) and overactive bladder (OAB) status, were recruited in Pennsylvania and North Carolina. Focus groups were conducted by trained female moderators and sessions were audiotaped. Participants completed an anonymous questionnaire containing validated items to determine the presence of UI and OAB. Audiotapes were transcribed and content was analyzed by two investigators to identify themes.

**Results:**

Twenty-four women participated (mean age 68 ± 13.4 years); most had UI (75%) or OAB (87.5%). Many women had difficulty in describing bladder health, and talked about bladder function, diseases or conditions, and control over the bladder. Four themes about toileting emerged: 1) cues/triggers/alerts women used to find and use toilets, 2) toilet cleanliness away from and at home, 3) toileting as a nuisance, and 4) situational awareness. Women described internal (e.g., sensation of heaviness) and external cues/triggers/alerts (e.g., walking by restrooms), and the trade-off between their concerns about public toilet cleanliness and the need to urinate. Some women expressed being irritated or annoyed about having to stop activities to urinate. Most women reported sitting on their home toilets, whereas, many hovered or stood over the toilet in public places.

**Conclusions:**

The information gained from this study will facilitate the development of relevant public health messaging and interventions to raise public awareness about UI, OAB, and bladder health with the aim to encourage women to seek help when symptoms are present.

**Electronic supplementary material:**

The online version of this article (10.1186/s12905-019-0740-3) contains supplementary material, which is available to authorized users.

## Background

Women’s bladder health has followed in the footsteps of women’s heart health by becoming a priority at the national level in the United States. The National Institutes of Health National Institute of Diabetes and Digestive and Kidney Disease (NIDDK) expanded its focus on girl’s and women’s urologic health by creating a research consortium to investigate multi-level factors, including behaviors necessary to prevent lower urinary tract symptoms (LUTS) in women [1, 2]. Better understanding about the reasons for and impact of women’s behaviors and how they may act as barriers or facilitators to bladder health in different environments (i.e., personal environment of home versus public environments that include workplace and social/community) is viewed as being important to prevent and manage two prevalent LUTS: 1) urinary incontinence (UI), and 2) overactive bladder (OAB), which includes urinary frequency, urinary urgency with and without urgency urinary incontinence [3, 4].

Because women live and work in various physical and socio-cultural environments, they may adapt their usual toileting behaviors or develop new ones either by choice or out of necessity. For example, some women may ignore the physiologic need to urinate and not use the toilet while they are at work due to limited breaks [5], limited number of available toilets, or lack of opportunity to leave work to urinate [4, 6]. As a result, women may develop a habit of pre-emptively voiding when the opportunity to urinate arises [7] or straining for voiding initiation [8]. Our objective for this study was to better understand what women know about bladder health and what they say about meeting urination needs.

## Methods

Focus groups were conducted between January and March 2015 in two locations in the United States: Chapel Hill, North Carolina and Philadelphia, Pennsylvania. The aim was to elicit women’s responses about what a healthy bladder meant to them and to identify behaviors they used to urinate at home and away from home. Administrators from the sites, an urogynecologic clinic (North Carolina) and two senior centers (Pennsylvania), provided access to potential participants and private conference rooms on the day of the focus groups. Senior centers are community centers where older adults engage in multiple social, physical and intellectual activities to help them remain healthy and independent. The Institutional Review Boards of the University of North Carolina at Chapel Hill and the University of Pennsylvania approved this study.

Women, regardless of their UI and OAB status, from ethnically diverse backgrounds aged 21 years and over who were not pregnant, and who could read and write English were eligible to participate. Women were recruited by displaying IRB-approved flyers in clinics and senior centers, email listserv, and by word of mouth. They were contacted again by email or phone call 24-h prior to the focus groups. Women who agreed to participate provided written informed consent prior to the focus group discussion.

Two trained female research team members (BR and DKN) moderated the focus groups and used a discussion guide with probing questions to facilitate participation. The discussion guide reflected major constructs from the Toileting Behaviors: Women’s Elimination Behaviors (TB-WEB) questionnaire: place preference for voiding, premature voiding, delay voiding, straining voiding, and position preference for voiding [9]. The research team revised the discussion guide that included seven interview questions with associated probing questions in consultation with a qualitative study design expert. An additional file contains the discussion guide, see Additional file 1. A research team member attended each focus group to obtain written informed consent, take field notes, assist with distributing refreshments and study materials, administer and collect questionnaires, and distribute $25 gift cards to the participants.

Women were told that the session would be audiotaped, but they were assured their identity would be kept anonymous. At the end of each focus group, they were asked to complete an anonymous questionnaire that elicited information on several demographic variables, and two validated instruments that are part of a series of questionnaires used in clinical practice to evaluate treatment effectiveness and impact of symptoms. The International Consultation on Incontinence Questionnaire-Short Form (ICIQ-UI SF) (four items) [10] is used to assess symptoms and impact of UI and the International Consultation on Incontinence Questionnaire-Overactive bladder (ICIQ-OAB) (eight items) [11] is used to assess symptoms and impact of OAB.

The audiotaped data were transcribed and then coded/analyzed using Atlas.ti7. Coding was an iterative process in which two investigators reviewed transcripts independently to develop coding schema and identify common themes. The investigators discussed the themes they identified and refined/collapsed those themes. The transcripts were re-reviewed and to the greatest degree possible, the investigators sought to define themes and coding schemes based on the input of focus group participants, as opposed to the structure of the focus groups, which can be influenced by the moderator, or existing frameworks, or definitions in the literature.

## Results

Twenty-four women (average age 68 ± 13.4 years) participated in focus groups that lasted approximately 60 min. Most women reported had UI or symptoms of OAB, see Table 1.

**Table 1:** Characteristics of focus groups’ participants

	Overall (*N* = 24)	North Carolina (*N* = 8)	Pennsylvania (*N* = 16)
	N (%)	N (%)	N (%)
Age in years (mean, SD)	68 (13.4)	56 (14.5)	74 (7.8)
Body mass index (mean, SD)	30.7 (9.4)	24.0 (4.0)	34.1 (9.6)
Race			
White	7 (29.2%)	6 (75%)	1 (6.3%)
Black	15 (62.5%)	2 (25%)	13 (81.3%)
Missing data	2 (8.3%)	0 (0%)	2 (12.5%)
Ethnicity			
Hispanic	1 (4.2%)	0 (0%)	1 (6.3%)
Non-Hispanic	21 (87.5%)	8 (100%)	13 (81.3%)
Missing data	2 (8.3%)	0 (0%)	2 (12.5%)
Urinary Symptoms			
Presence of UI	18 (75%)	5 (62.5%)	13 (81.3%)
Presence of OAB	21 (87.5%)	6 (75.0%)	15 (93.8%)

*SD* Standard Deviation

Animated talking, often interrupting or talking over each other, was a frequent behavior. Participants expressed support for women with UI, with one woman saying, “we are not embarrassed with it.” Another woman said, “it’s not an old-lady, you know, syndrome. It can happen to anyone.” She also said that it was as if they belonged to, “a secret club.”

The moderators asked the women what bladder health meant to them. Many women had difficulty in describing it. They talked in general terms such as: 1) how the bladder functions; 2) the presence or absence of infection or disease; and 3) control over the bladder. For example, when the women talked about bladder function, they mainly talked about the bladder’s emptying function, e.g., emptying all the urine stored in the bladder, having a blockage that did not let urine empty, getting rid of wastes, and not having to use the bathroom frequently. The women also talked about bladder health, in terms of the presence or absence of symptoms. One woman mentioned, “burning,” and another woman said, “pain in her back.” A woman talked about having bladder cancer in the context of bladder health because a family member had had bladder cancer.

Most women talked about bladder health as control, i.e., the ability to control or hold urine and keep it from emptying accidently or unintentionally. One woman said bladder health was, “pretty much control.” Control under certain conditions was also discussed such as drinking large amounts of water, holding urine and waiting too long to go to the toilet, and being able to hold urine longer at certain times compared to other times. Women also discussed bladder control challenges when taking medications, especially diuretics for blood pressure.

### Toileting behaviors’ themes

Four themes emerged: 1) cues/triggers/alerts for women to seek and use toilet facilities to empty their bladders, 2) cleanliness matters, 3) toileting as an irritant and a nuisance, and 4) situational awareness, see Table 2.

**Table 2:** Identified themes and direct quotes from participants

**Theme: Cues/triggers/alerts**	
*“It’s a mental cue. You walk past a water fountain, a bathroom, you know, anything that correlates with going, like a flow.”* *“It’s almost like I think, OK, I’m going to be busy for an hour, now perhaps I should go to the bathroom now, when I see one.”* *“Oh, WOW! I just have kind of a pressure kind of feeling. You know, and it’ll start off small, and if you wait too long then it’s a real pressure and you feel like you have to really hurry.”* *“And running water. Yeah, that’s when it— and somebody else have to go, then you say, I better go too. [Chuckling] Running water (make it come down?) too.”*	
**Theme: Cleanliness matters**	
*“No, I was just going to say about, you didn’t mention this, but when you sit on the toilet like that you can get an infection.”* *“I don’t have any problem, long as the bathroom is there, I have to go, use.”* ***“*** *If I see what the position the bathroom is, most of the time I don’t want to sit down because if the bathroom is not really clean, yeah, I just have to be very careful. Some places they put the paper, you know, the one you can put and cover it—”* *“—it’s the cleanliness of it. But here’s a thought I had, I’ve thought about this, I thought, if you’re sitting on the seat, I wonder how many germs, really, from the last person, or how long those germs survive on the seat.”* *“I was taught that [to hover], but my, since my legs got so bad, I can’t do that standing up and squat. So I have to pad it” (*i.e.*, the toilet seat), “I pad it all around it, and I sit to the edge.”* *“Oh, I do that, because I have company and every now and then I will go up there and spray and wipe. Especially if there’s a lot of folks.”*	
**Theme: Toileting as an irritant and nuisance**	
*“I find having to go to the bathroom a pain in the neck. I find it like, Oh, god, I’ve got to go to the bathroom!”* *“Sometimes on days when I’m like super hydrated and I feel like I’m going to the bathroom more often and it’s kind of, it’s kind of a pain in the rear end, I will, when I finish I like, Oh, I feel good, I feel better, I wish that would last longer.”* *“I think it was that. I think it was a lot of that. Just enough to take the pressure off to then to get on to the next thing.”* “*… I don’t feel like I have to empty all the way, just enough to where you feel like it’s going to stop and you’re good.”*	
**Theme: Situational awareness**	
*“Oh, I would almost probably always stalk out where the bathroom was. Always.”* *“—there’s certain toilets I will not use. One is in an airplane. Unless it’s absolutely necessary. I will hold it for hours before I will—and I’m bad about that.”* *“Or if I hear somebody and it feels like they’re—sounds like they’re pouring a five gallon bucket, I’m like, Good lord, how much did you drink? I perceive other people, I have noticed other people going, so I worry that they are paying attention to me—which they probably aren’t.”* *“You don’t, you know, you lose all sense of kind of modesty when you have to, you just got to go. You’ve got to go. But, yeah, depending on the place, too, whether it’s a crouch or a hover.”* *“So when I was talking about worrying about what people think, it’s when I’m at work and I know that there’s a coworker in the bathroom.”* *“there’s certain toilets I will not use. One is in an airplane. Unless it’s absolutely necessary.”* *“You just excuse yourself from the situation, and go do what you need to do. Because a lot of times you don’t want people to know”, “God, she’s going to pee how many times now?”*	

### Cues/triggers/alerts

Cues/triggers/alerts could be both internal and external to the women. When asked about what alerted them to urinate, most talked about internal cues such as feelings of fullness, urge or urgency, heaviness, pressure, and tightness. A woman noted she felt the urge to urinate when changing positions from sitting to standing. Another woman said that the *thought* of needing to use the toilet preceded the actual sensation of the need to urinate. A participant connected the amount of water she drank with her frequent urination.

The women also talked about external (i.e. auditory, visual, sensing nearness/farness of toilets, and social) cues such as hearing water run or splash, pumping gas into a car, walking into a restaurant, walking by a water fountain or bathroom and other women saying they needed to use the restroom. One woman said that when she went through the door to her house, she suddenly needed to go to the toilet, even if it was a small amount. She attributed it to, “a brain thing.”

### Cleanliness matters

While describing the decision to use a toilet while away from home, many women described a trade-off between concerns about cleanliness and the need to urinate. One woman mentioned that non-flushable portable toilets and airplane toilets concerned her, and that she would use them only in extreme emergencies. Another woman acknowledged that while, “nasty public toilets” are “gross,” she still used them because, “the discomfort is not worth it to me.”

The women also discussed techniques they used to ameliorate their concerns about cleanliness. Hovering/crouching or standing over the toilet, lining or padding the toilet seat with tissue paper, tissue toilet seat covers, or paper towels, pushing urine out to empty the bladder quickly, not waiting to empty the bladder completely, and cleaning and wiping the toilet seat before sitting on it were commonly mentioned techniques.

The cleanliness of and worry about cleanliness of public toilets were prominent factors for women deciding to sit or hover over toilets or delaying voiding until they could get home. Most women reported either hovering or standing over a public toilet to urinate. Some older women needed to use the handicap bars to use the toilet and no longer could hover over the toilet due to weakness in their thighs, but they did not want to sit on toilet seats to urinate. Two women used a men’s room at gas stations to avoid using dirty toilets in women’s restrooms. Women compared their cleanliness ratings for different public toilets stating which fast food places, stores, and highway rest stops that regularly had clean restrooms. While some women wondered and worried about germs on public toilet seats, other women said that, they did not worry about being exposed to germs. One woman worried about splashing, i.e., fluid in the toilet splashing up onto her body and that she set a 5-s rule for sitting on public toilets to get, “fewer germs.” Another woman had concern about germs from toilets, but thought that, “not all germs are going to kill me.”

Few women talked about cleanliness of their toilet at home, but several older women in one focus group discussed different commercial products used to clean or sanitize their home toilets, even when they did not have guests or others using them. One woman said that even though no one else used it, “You still got to clean that toilet”.

### Toileting as an irritant and a nuisance

Many of the women found urination to be a nuisance, because it caused them to stop what they were doing to urinate, especially in a public place or when they were engaged in enjoyable activities, like shopping or being with friends and family. A woman said, “You know, it’s like an irritant to me almost.” Some women held urine intentionally until they got home. Other women, when they were in public places, wanted to get urination over quickly, and sometimes did not sit long enough to completely empty their bladders. One woman said, “… but I think I just do enough where I probably don’t completely empty out.”

### Situational awareness

For some women, traveling presented concerns other than cleanliness of toilets. A woman said, “I hate to lock the door anyway, because I’m scared if I get in there I can’t get out.” When talking about airplane travel, women reported avoiding the toilets on board. and expressed dislike of the smallness of the toilet compartment and knowing that strangers used the same toilet.

Driving on highways also presented a challenge to some women. One woman talked about a time when she was driving a long distance, stopped at a gas station in the rain, and began to pump gas. She said, “It was like once the gas started, I had the urge to go to the bathroom. I had to sit on the curb and I wet myself.”

Women talked about the best places to stop to go to the toilet while traveling by car. Several women talked about limiting fluids before traveling or wearing absorbent products while on a car trip. Some older women who went on bus trips complained about the small size of toilet cubicles on the buses and one woman said, “It’s ain’t nothing but a little cubby hole.”

Several women differentiated behaviors they used in public places from those they used in familiar (at home, or at family and friends’ homes) surroundings. Most said they usually sat on the toilet, felt more relaxed or comfortable than in public places, and usually waited to completely empty their bladders while urinating at friends’ homes, as they would at home. Discussion about public toilet use led some women to reflect on what other women in the restroom might think. One woman talked about how long it took her to empty her bladder and said, “And then you wonder what those people think you’re doing.” She laughed and then said, “a couple of times I was caught in that situation where I felt a little self-conscious. And then my mind goes to, I don’t know these people, I’ll never see them again in my life, so why am I worried?”

## Discussion

The women participating in the focus groups were willing to talk about bladder health and toileting behaviors. They had difficulty in describing bladder health. This is not a surprising findings given that until recently no bladder health definition existed, [12] and there has been little emphasis by researchers or clinicians on the importance of bladder health as a component of overall health [13]. In this study, most women viewed bladder health in terms of the bladder’s emptying function, absence of infection or disease, and control over urination. Despite their difficulty in describing bladder health, women readily described behaviors they used to manage urination. They were frank and unabashed in discussing personal experiences of being incontinent, about wearing pads or panty liners, and having urinary urgency or uterine or bladder prolapses with a group of women they just met. Some women used humor when talking about their bladders. During most focus groups, there was laughter and talking over each other as women volunteered personal information about near or actual episodes of urine loss. The use of humor when talking about UI has been described as, “testing the social waters for risks to self-esteem or safety in disclosure” [14].

Women used terms like, “pressure”, “fullness”, “heaviness”, “urge to go”, and “indicator feeling.” These are similar to categories created by Das and colleagues (i.e., “urgency, fullness, pressure, tickle/tingle, pain/ache, heavy, normal, intense, sudden, annoying, uncomfortable, anxiety, unique somatic,” [15] p. 161). Understanding the language women use in describing sensations related to their bladder and its function is important for healthcare providers to know and potentially use when eliciting information from women during screening, clinical assessment, and education. This information may help clinicians to discriminate the urinary urgency sensation in women who have urinary urgency from women who do not [16].

Earlier a focus group conducted with 25 women explored the relationship between “conscious decision-making processes and bladder sensation to determine the need, time, and place to void” [17]. The authors found that women are often prompted by cues or signals other than the urge sensation to urinate, although awareness of bladder volume was the key final factor in decision to urinate. Our findings were similar to Harvey et al.^17^; many women talked about making decisions to urinate because toilets were available, it was a convenient thing to do in relation to other activities, or other women suggested using or were using the restroom. Some women anticipated the need to urinate before the need itself occurred by considering the amount of fluid they had ingested, the distance and times to the next restroom, and upcoming work and social activities. Women considered a range of factors beyond bodily functions when deciding to use the toilet to urinate. A combination of physiologic, cognitive, psychosocial, and environmental factors appears to play a role in toileting behaviors. The cumulative effect of these behaviors and environmental effects on risks of developing UI or OAB over the lifecourse is not known.

Many women worried about cleanliness of public toilets. This worry can start in childhood, with children disliking unpleasant smells or experiencing bullying in school restrooms [18], and continue throughout adolescence [19] and adulthood. As other authors noted, women must attend to several biologic functions: urination, defecation, and menstruation, often in public places. The sight and odor of the products of these functions can elicit feelings of disgust and generate an unconscious disease-avoidance behavior [20].

Variation in perceptions about cleanliness existed with some women saying that they did not worry about cleanliness of public toilets while others had considerable worry and used different toileting positions away from home. In a recent survey, 98% of employed women (average age 48 years) reported sitting on the toilet often or always when at home, while only approximately 68% sat on toilets often or always when away from home [21]. Young women between 18 and 25 years old also reported worry about toilet cleanliness (87%); approximately 47% pre-emptively voided, and 67% delayed voiding due to busyness [22]. These researchers found that specific toileting behaviors were associated with LUTS.

Further research is needed to better understand the relationship between toileting behaviors and UI and other LUTS. Two unidirectional relationships may be possible. First, women change their behaviors to adapt to LUTS and second, behaviors women use to urinate play a role in LUTS development and progression. A bidirectional relationship may also exist; thus, more research is necessary to explore the underlying mechanism between behaviors women use and disease development and prevention. A surprising finding was that some women intentionally did not fully empty their bladders when they urinate, usually when they were away from home. Further investigation is needed about the prevalence of this behavior in the adult female population and its impact on bladder health.

Women with and without UI and OAB participated in the focus groups and no attempt was made to correlate behaviors with urinary symptoms due to the exploratory nature of the study. Women with UI and other LUTS may engage in different behaviors than women who do not. Better understanding the role of behavior and environment in the natural history of different LUTS, especially UI and OAB, is vital to prevention and treatment of these prevalent symptoms.

## Conclusions

Understanding how women talk about their bladders and the behaviors they use to manage urination will assist researchers in identifying factors that may affect bladder health. In our focus groups, women were willing, often eager, to talk about their bladder symptoms as well as their toileting behaviors despite the sensitive nature of the topic. The information gained from this study will facilitate the development of relevant public health messaging and interventions to raise public awareness about LUTS. More research is needed about the relationship among bladder health, toileting behaviors used in different environments, and the development and progression of UI and other LUTS in women.

## Additional file

Additional file 1: Discussion guide. Questions used during focus groups. (DOCX 16 kb)

## Abbreviations

BMI: Body mass index
ICIQ-OAB: International Consultation on Incontinence Questionnaire-Overactive bladder (eight items)
ICIQ-UI SF: The International Consultation on Incontinence Questionnaire-Short Form (four items)
LUTS: lower urinary tract symptoms
NIDDK: The National Institutes of Health National Institute of Diabetes and Digestive and Kidney Disease
OAB: overactive bladder
SD: Standard deviation
TB-WEB: Toileting Behaviors: Women’s Elimination Behaviors
UI: urinary incontinence (UI)
