# Supplementary material for: “A secret club”: focus groups about women’s toileting behaviors
Source: BMC Womens Health. 2019 Mar 7;19:44. doi: 10.1186/s12905-019-0740-3 (PMC6407194; doi:10.1186/s12905-019-0740-3)
Supplement: Supplementary file 1 — Discussion guide. Questions used during focus groups. (DOCX 16 kb) [file 12905_2019_740_MOESM1_ESM.docx]

**Additional file 1. Focus group questions**

1. What does the phrase “bladder health” mean to you?

Probes:

1. Is there anything else that comes in mind?
2. What mental image do you have, if any, when you hear the phrase bladder health?
3. What alerts you that you need to use the bathroom?
   1. What cues do you experience that cause you to go to the bathroom?
   2. For example, some women may say they feel their bladder feels full, or maybe they walk by a bathroom and feel like they have to go.
   3. Some women may feel a strong urge like they cannot hold back urine.
4. Think about when you are using the bathroom. What language do you use to describe using the bathroom?
   1. For example, some women may think about it in terms of releasing urine, getting rid of the urge to urinate, etc…
   2. What does the phrase empty your bladder mean to you? (i.e., what goes through your mind when you hear the phrase “empty your bladder?
5. Does knowing where bathrooms are located affect your plans to go places?”
   1. Does this affect how long you feel like you can stay at that place?”
   2. Does this affect your travel plans? For example, driving long distances, flying on airplanes, taking buses?
6. Does having to use a public bathroom affect your decision to go to the bathroom?
   1. Can you tell me more about that?
   2. Does having to use a public bathroom affect your plans to go out? If so how?
   3. Does having to use a public bathroom change how you behave as compared to when you are using the bathroom at home or at work?
7. How do you feel about using a public bathroom?
   1. How, if at all, does it affect your behavior? (Does it affect the position you use to go to the bathroom?)
   2. Are there times when you wait until you find a different bathroom to go? Can you tell us more about that?
   3. Do you hurry or slow down your stream of urine to keep others from hearing you?
8. Is there anything you’d like to discuss that we haven’t yet talked about?
